# Treatment burden experienced by patients with obstructive sleep apnoea using continuous positive airway pressure therapy

**DOI:** 10.1371/journal.pone.0252915

**Published:** 2021-06-07

**Authors:** Michael S. H. Chou, Natasha C. H. Ting, Nicole El-Turk, Zinta Harrington, Claudia C. Dobler

**Affiliations:** 1 Department of Respiratory and Sleep Medicine, Liverpool Hospital, Sydney, NSW, Australia; 2 South Western Sydney Clinical School, University of New South Wales (UNSW), Sydney, NSW, Australia; 3 Institute for Evidence-Based Healthcare, Bond University and Gold Coast University Hospital, Gold Coast, QLD, Australia; University of Auckland, NEW ZEALAND

## Abstract

**Background:**

Little is known about the treatment burden experienced by patients with obstructive sleep apnoea (OSA) who use continuous positive airway pressure (CPAP) therapy.

**Participants:**

18 patients (33.3% males, mean age 59.7±11.8 years) with OSA who use CPAP therapy were interviewed.

**Methods:**

Patients treated with CPAP for OSA at a tertiary hospital outpatient clinic in Sydney, Australia, were invited to participate in an interview in person or via phone. Semi-structured interviews were used to explore the treatment burden associated with using CPAP. The interviews were recorded, transcribed, and analysed using NVivo 12 qualitative analysis software.

**Results:**

Four categories of OSA-specific treatment burden were identified: healthcare tasks, consequences of healthcare tasks, exacerbating and alleviating factors of treatment burden. Participants reported a significant burden associated with using CPAP, independently of how frequently they used their device. Common sources of their treatment burden included attending healthcare appointments, the financial cost of treatment, lifestyle changes, treatment-related side effects and general discomfort.

**Conclusions:**

This study demonstrated that there is a significant treatment burden associated with the use of CPAP, and that treatment non-adherence is not the only consequence of treatment burden. Other consequences include relationship burden, stigma and financial burden. It is important for physicians to identify other negative impacts of treatment burden in order to optimise the patient experience.

## Introduction

Obstructive sleep apnoea (OSA) is a common chronic condition [1], affecting an estimated 9% to 38% of the adult population globally [2]. Patients with OSA experience episodes of sleep disruption due to recurrent complete or partial airway obstruction, causing snoring [3]. This can further cause excessive daytime sleepiness, depression, and inability to concentrate [4], which can have a detrimental effect on the patients’ quality of life.

Continuous positive airway pressure (CPAP) is the mainstay of OSA treatment, together with exercise and dietary management in overweight patients [5]. Although many studies have shown CPAP’s effectiveness in improving excessive daytime sleepiness, depression, and ability to concentrate [6–9], patient non-compliance with CPAP treatment is an issue, with a systematic review reporting an overall non-adherence rate of 34% among patients with OSA [10]. CPAP adherence is commonly defined as a minimum use of 70% of all nights with at least 4 hours of nightly usage [11]. Previous interview studies investigating patients’ reasons for non-adherence to CPAP therapy have identified general discomfort using CPAP, CPAP-related side effects, treatment affordability, restriction to intimacy, and stigma as barriers to adherence [12–14].

Whilst previous studies have examined different aspects of challenges and reasons behind non-compliance that patients with OSA on CPAP therapy encounter, no study to date gives a comprehensive overview of all the elements of treatment burden experienced by patients with OSA using CPAP therapy. Treatment burden is a relatively new concept, describing the patient-perceived workload of managing their illness(es) such as the effort of learning about their disease(s) and treatments, making lifestyle changes, and arranging appointments with healthcare professionals [15, 16]. Treatment burden also addresses the subjective impact of healthcare-related tasks on patients [17, 18]. For example, patients with cognitive deficits may struggle more with managing medications compared to those without cognitive deficits. The former group may, therefore perceive a higher treatment burden associated with the same treatment and may consequently be non-compliant with treatment. Treatment can also have significant financial, social, and psychological burdens [16, 19].

Non-adherence to treatment is only one of the possible consequences of a high perceived treatment burden. Other consequences of high treatment burden include the impact on one’s social life and finances due to stigma and cost of treatment [16]. Therefore, even in patients who are adherent to CPAP therapy, there may be a significant burden hidden from clinicians.

Existing treatment burden frameworks were developed based on chronic diseases such as chronic obstructive pulmonary disease (COPD), diabetes, cardiovascular diseases, and cystic fibrosis [16, 18–21]. Hence, not all proposed aspects of treatment burden are applicable to OSA and CPAP therapy.

The goal of the study was to evaluate the whole treatment burden experienced by OSA patients who are using CPAP therapy, including but not limited to the burden associated with the device use. The results can be used to inform discussions between clinicians and patients on treatment burden with the goal to alleviate this burden on patients.

## Methods

### Setting, study design and participants

This qualitative study utilised semi-structured interviews and an inductive-deductive approach to explore the treatment burden perceived by patients with OSA using CPAP. The deductive approach was the development of an OSA-specific treatment burden framework and an interview guide based on (i) themes highlighted in the literature exploring patient experiences with CPAP therapy and (ii) existing treatment burden frameworks for other chronic diseases. The themes of treatment burden addressed in the OSA-specific framework we developed were then used as pre-determined categories for coding. The inductive approach involved adding new themes found through the interviews into the existing framework of OSA-specific treatment burden. The interview guide was refined after two pilot interviews, which resulted in minor changes in the sequence and wording of questions.

We used purposive sampling (also known as selective or subjective sampling) to select participants who were willing to talk about different aspects of the burden of treatment they experienced. Our study did not aim to establish what percentage of patients with OSA suffered from a specific treatment burden but to elicit and understand any possible areas of treatment burden that patients with OSA could potentially experience. Patient recruitment was performed in the outpatient respiratory and sleep clinic of Liverpool Hospital in Sydney, Australia. Patients with OSA on CPAP therapy were identified from the clinic’s outpatient list and were contacted via phone. Patients were either interviewed in person or via the phone. Patients were eligible if they were 18 years and older and had been on CPAP treatment for at least four weeks. Patients were not eligible if they could not communicate in English without an interpreter or if they were using CPAP for a diagnosis other than OSA. Ethics approval was obtained from the South Western Sydney Local Health District Human Research Ethics Committee.

### Data collection and analysis

Interviews explored all burdensome aspects of CPAP therapy from the patient’s perspective by using open questions and were conducted until data saturation occurred. Semi-structured interviews allowed participants to guide the conversations and enabled an in-depth exploration of themes mentioned by the participant. Pre-determined topics of interest included in the interview guide were brought up by the interviewer and further explored.

All interviews were recorded and transcribed. Interview transcripts were uploaded into NVivo 12 qualitative analysis software for data analysis and coding. Four interviews were independently coded by two other researchers using the coding framework developed by the principal investigator. Discrepancies between the coding were critically evaluated and discussed until a consensus was reached. Specific suggestions were then incorporated into refining the coding framework.

### Definitions

The severity of sleep-related breathing disorders is measured using the participant’s apnoea-hypopnea index (AHI) or respiratory disturbance index (RDI). This paper adopted the original definition proposed by the American Academy of Sleep Medicine Task Force in 1999, which defined mild, moderate, and severe OSA as an AHI or RDI of 5–14, 15–30, and >30 events per hour respectively [22].

### Quantification of treatment burden

Patients quantified and rated different aspects of treatment burden on a Likert scale from one to five, with five indicating the highest possible burden, and one indicating no burden at all. If patients had not given a quantitative rating, a score was assigned by the investigators based on interview responses to that specific theme.

## Results

### Participant’s demographics

Between April and August 2019, 36 patients were invited to participate in the study, and 19 patients provided informed consent to participate in this interview study. The 17 potential participants who were approached but did not consent to participate in the study were not willing to stay for the interview after they had attended a clinical appointment at the outpatient clinic. Of the 17 potential participants, two agreed to participate in a phone interview later; however, they could not be successfully contacted afterwards despite multiple attempts.

Interviews ranged in duration from 15 minutes to 1 hour (mean duration: 31 minutes). One interview was excluded when it became apparent that the participant was using CPAP therapy for a diagnosis other than OSA. Most of the participants had moderate to severe OSA (16 out of 18 patients) with their AHI well-controlled on treatment (mean AHI on treatment: 4.3 episodes/hour). Of the 18 participants, 17 suffered from daytime somnolence, and one patient suffered from disrupted sleep due to heavy snoring, which also put a strain on the relationship with his partner. The mean duration of CPAP treatment among the participants was 33 months. Three patients initially ceased the use of CPAP many years ago but restarted treatment recently. Participant demographics were summarised in Table 1 below.

**Table 1:** Basic participants demographics.

Patient characteristics	n = 18	Percentage (%)
Age:		Median: **61** years
• Mean: **59.7 ± 11.9** years		Range: **41–85** years
Sex:		
• Male	6	33.3%
• Female	12	66.7%
Working status:		
• Not working	12	66.7%
• Working	6	33.3%
Interview mode:		
• Phone interviews	10	55.6%
• Face-to-face interviews	8	44.4%
Time since CPAP diagnosis made:		
• Less than 1 year	3	16.7%
• 1 to 2 years	5	27.8%
• 3 to 5 years	5	27.8%
• 6 to 10 years	2	11.1%
• 11 years or more	3	16.7%
Duration of CPAP treatment:		
• 1 to 6 months	4	22.2%
• 7 to 12 months	6	33.3%
• 13 to 24 months	1	5.6%
• 25 to 60 months	5	27.8%
• 61 months or more	2	11.1%
Machine status:		
• Purchased	7	38.9%
• Rented	4	22.2%
• Government-funded	7	38.9%
	n = 16*	**%**
OSA severity before treatment (n = 16*):		
• Mean AHI: **40.4 ± 26.9** events/hr		
• Median AHI: **30** events/hr		
• Range: **10.1–96** events/hr		
Patient’s OSA severity (sorted according to the original definition proposed by the American Academy of Sleep Medicine Task Force in 1999):		
• Mild (AHI of 5–14)	2	12.5%
• Moderate (AHI of 15–30)	7	43.75%
• Severe (AHI > 30)	7	43.75%
OSA severity on treatment (n:17**)		
• Mean AHI: **4.3** events/hr		
• Median AHI: **1.2** events/hr		
• Range: **0–32.9** events/hr		

*Two participant’s AHIs before treatment was not available since their original diagnostic sleep study was missing;

**One patient has no follow-up data available.

### OSA-specific treatment burden framework

An OSA-specific treatment burden framework was created (S1 Appendix) using elements of treatment burden from existing frameworks with the addition of themes discovered through the study interviews. Four central themes were identified: healthcare tasks, consequences of these healthcare tasks, factors that alleviate treatment burden, and factors that exacerbate treatment burden. A summary of the treatment burden framework has been provided in Table 2.

**Table 2:** Simplified treatment burden framework for patients with OSA using CPAP.

Central themes	Main themes
Healthcare tasks	• Sleeping with CPAP
	• Maintaining Lifestyle changes
	• Attending healthcare appointments
	• Learning relevant knowledge regarding CPAP and OSA
	• Travelling with CPAP
	• Equipment maintenance
Consequences of healthcare tasks	• Non-adherence
	• Financial burden
	• Relationship burden
	• Treatment-related side effects
	• Stigma
Factors that exacerbate treatment burden	• Culture factors
	• Financial difficulties
	• Poor access to healthcare resources
	• Little perceived health-benefits from treatment
Factors that alleviate treatment burden	• Good relationship with healthcare providers
	• Substantial health benefits from treatment
	• Alleviate existing tension in relationships
	• Support and help from one’s spouse, family members and other carers

#### 1) Healthcare tasks

Among all the tasks that participants needed to perform to take good care of their OSA, they struggled the most with adjusting to sleeping with CPAP and acquiring the relevant knowledge about how to use CPAP. Equipment maintenance was very burdensome for some participants while not being a problem at all for others.

*Sleeping with CPAP*. Getting used to sleeping with the machine was perceived the most burdensome, with 12 participants having a burden level of four or above. All patients reported experiencing problems with the CPAP equipment, such as air leakage, uncomfortable mask fit, excessive machine-generated noises and excessive water condensation in the mask due to the humidifier (rainouts). At higher air pressure, six participants even reported experiencing feelings of suffocation. Among all these problems, air leakage was the most frequently brought up and discussed. Many participants attributed the problem to a poorly fitted mask, which left gaps for air to be leaked. These poorly fitted masks were also uncomfortable to wear, making it even harder for patients to acclimatise to the use of CPAP. Four participants had to change their usual sleeping positions to sleeping on their back, to avoid air leaking from the mask. This was especially burdensome for patients who suffered from back pain. Several interviewees reported that they had to negotiate sleeping arrangements with their spouses (i.e. sleeping in separate bedrooms) due to machine-generated noise and air leakage from the mask, with leaked air occasionally blowing onto the spouses’ face, interrupting their sleep.

Rainout, whereby the condensation can flow to the eyes and nostrils, has been well documented in the literature and was mentioned by several participants. The condensation can flow into the nostrils and eyes. Participants reported being woken up because of rainouts and had to wipe it off, which had a significant negative impact on the participant’s sleep and burden:

*I found that when I put water into the water tank*, *I have a lot of condensation on the inside of the mask*, *which makes it ten times worse*. *I will have to get up in the middle of the night and dry my mask and face and everything because it leaks*. *(Heidi*, *49-year-old female)*

The quality of air supplied by the machine was also mentioned in several interviews. Some participants described the smell of the air supplied as “*funny”* and *“not fresh”*, which made it hard to get comfortable. The air temperature supplied by the machine was also brought up:

*Because the humidity of the airflow is high*, *its temperature is also high*. *So*, *trying to breathe in the warm air*, *(especially) early in the morning*, *it’s hard to get comfortable in it*. *It’s hard (to get used to)*, *and this doesn’t feel fresh*. *(Luke*, *41-year-old male)*

Many participants also complained about problems with the mask strap. Two participants who had long hair voiced that their hair would occasionally adhere to the Velcro part of the head strap, and removal of the hair from the Velcro could be painful. Six participants also disliked the indentation in the hair left by the strap, which needed extra effort when getting ready in the mornings:

*I have to blow dry my hair every day*, *which I never had to do before… It [the head strap] squashes it all…*. *I’m busy*, *and I work*, *(and) I don’t necessarily have the time to be doing my hair from scratch every day*. *(Belinda*, *71-year-old female)*

Two participants also complained about pain and bruising caused by the head strap:

*The strap used to hurt me at the back of my head*. *It was very sore all the time*. *(Stephanie*, *66-year-old female)*

*It [the head strap] would rub on the back of my neck and irritate me all the time… and because of that*, *it was bleeding all the time*. *(Sophie*, *47-year-old female)*

*Attending Healthcare Appointments*. Burdens associated with healthcare appointments included transport, parking cost, opportunity cost, waiting times, the burden on carers, and getting ready for healthcare appointments. Several participants felt they were a burden to their family members who drove them to these clinical appointments, with one patient stating:

*Normally*, *my husband comes and drops me off*, *and then he will go home and sleep*. *(This is) because he works the night shift*, *he can only sleep when I finish here in the hospital*. *(And) I am keeping him awake when he needs to drive me here*. *(Karen*, *46-year-old female)*

*Travelling with CPAP*. Different types of power points in different countries meant patients had to buy power adapters to use CPAP in foreign countries, which was described as “*a hassle*”. Another participant experienced a significant burden in using CPAP when travelling to countries where there was limited access to clean water for use in the CPAP humidifier:

*You would not use tap water because they [sic] are not treated*. *We used bottled water*. *But even with bottled water*, *I am also not very comfortable with using it*. *Because in some shops*, *they refill it [used water bottle] with tap water and reseal it*. *That is very annoying*, *because what if we put the wrong water into the water tank*? *(James*, *66-year-old male)*

*Equipment Maintenance*. Five interviewees regarded machine cleaning as a burdensome task. Reasons for a high level of burden included lack of time due to a busy schedule, lack of required knowledge, and the complexity of the task, with one patient stating:

*It [machine cleaning] is quite a bit of a hassle because there are certain things that you can’t immerse in water and other things you can…that’s kind of like a mild irritation*. *(Belinda*, *71-year-old female)*

*Maintaining Lifestyle Changes*. Seven participants tried to exercise regularly to lose weight and improve their OSA. For others, common reasons for not exercising included busy schedules, the large effort required to exercise, and self-reported comorbidities such as arthritis and COPD. For those who tried to exercise regularly, more than half assigned a burden level of four or above for exercising (S2 Appendix). This burden was attributed to busy daily schedules and the huge effort required to exercise:

*I tried to (exercise regularly)*. *But it is hard to exercise because I am overweight*. *You get tired… and you can’t be bothered*. *You know you’ve been at work all day… and you cook today*, *and you don’t want to go for a walk because it’s dark*, *it’s cold*, *and you just can’t be bothered*. *(Sophie*, *49-year-old female)*

#### 2) Consequences of healthcare tasks

Consequences of healthcare tasks included non-adherence, financial burden, relationship burden and stigma. A few participants reported having experienced stigma due to their CPAP use. Maybe surprisingly in this study setting, participants struggled the most with the financial burden of using CPAP, even though the government subsidised many of the CPAP machines and clinic appointments were free of charge.

*Non-adherence*. Seven participants used CPAP less than 70% of all nights (for at least 4 hours per night). For many participants, their use of CPAP was interrupted by having a cold, hay fever or symptoms such as cough and blocked nose. Some interviewees stated that the repeat application of the mask during the night had led to non-adherence:

*When I get up to the toilet (in the middle of the night) and I need to put it on again after I come back (from the toilet)*, *that’s quite a hassle*. *Sometimes I don’t put it back on*. *(Gary*, *62-year-old male)*

*Financial Burden*. For interviewees who qualified for government subsidies and obtained the machine for free, half were still burdened by the maintenance cost of the machine, since the subsidy did not cover the cost of the mask and machine replacement parts. Among participants who did not receive any subsidy, many chose to rent the machine. Several participants had to cut back other expenses, to cope with the high financial cost of the CPAP machine and a gym membership. Two participants had to reduce money spent on their children:

*I have to cut back the expenses on the kids* … *I cut off Foxtel and other channels* … *they don’t even have computers and phones*. *(Karen*, *46-year-old female)*

*Stigma and Relationship Burden*. The majority of the participants did not feel stigmatised when using the CPAP machine. Participants who experienced stigma had few or no friends or family members who were also using the machine, and they felt that they were abnormal and being judged by others. One participant who had used the machine in Australia and India compared the experience:

When I was overseas, *everybody comes in and asks me*, *“Why are you using it*?*”*, *“What is this [machine]*?*”*,*” What is that thing sitting next to your bed*?*”*, *and then I feel embarrassed*. *But in Australia… in my family*, *other people are using it too*, *so I feel more comfortable and that I was not alone*. *(James*, *66-year-old male)*

Four participants felt embarrassed using the mask in front of their bed partners. Some participants expressed they felt less attractive with the mask, while some perceived the mask as a barrier to intimacy since the mask and hose interfered with intimate physical contact.

#### 3) Factors that exacerbate treatment burden

*Little Perceived Health Benefits from Treatment*. Many participants consciously weighed the treatment benefits versus the downsides of treatment, including adverse effects and treatment work. Participants who experienced little perceived treatment benefit found that the treatment did not warrant the discomfort during sleep and hence had less motivation to continue treatment. Interviewees stated:

*Why didn’t I use it*? *Because part of me didn’t feel like it made much difference*. *Also*, *I felt very claustrophobic when using the machine and having the mask on*. *(Olivia*, *63-year-old female)*

*It can be a burden because it’s not helping*. *I have tried and done all these tests… but I know I am still snoring*, *and I am suffering because of it*. *(Heidi*, *49-year-old female)*

*Cultural Factors*. Several participants expressed difficulty in losing weight due to their culture’s cuisine:

*It’s really hard to lose weight…Every week my family gets together*, *and there is food everywhere… For my background*, *we eat lots of carbohydrates*. *(Karen*, *46-year-old female)*

*Poor Access to Healthcare Services*. Some participants travelled two hours to get to the hospital. A participant voiced how the long time intervals (6 to 12 months) between appointments had slowed down her progress with CPAP therapy:

*When I first had the machine… There’s no one to ring and say*,*” the machine is doing this*, *what am I supposed to do*?*”* … *I have to wait for the next appointment to ask the doctor all my questions*. *But I will forget my questions because I was having trouble with something three months ago*. *(Sophie*, *49-year-old female)*

A number of participants expressed the need for a CPAP user support hotline, which would enable them to obtain advice between scheduled physician appointments. None of the participants who mentioned the need for a support hotline was aware that appointments at a specialist nurse clinic would have been available at short notice in the study hospital to assess problems with the CPAP equipment.

#### 4) Factors that alleviate treatment burden

For many, support from their spouse or family members was crucial in easing their burden. Some provided financial assistance, while others helped the patient with transport to clinical appointments. The presence of supportive spouses motivated some patients to use CPAP, as they were reminded to put on their mask before sleep and were relieved of any insecurities regarding their appearance with a CPAP mask, with one participant stating:

*I did feel a bit embarrassed (when I first had the mask) … I felt (more) comfortable after my partner told me that my health is more important than what I looked like*. *(Chris*, *47-year-old male)*

Many participants expressed how the cessation of their snoring had improved their spouses’ and other family members’ quality of sleep. This subsequently improved their relationship with these family members, which motivated them further to adhere to the treatment. Huge patient-perceived treatment benefits, including refreshing sleep and cessation of snoring, were the main motivators for patients to use the machine. Some participants believed that they would stop breathing altogether without CPAP, which motivated them to continue treatment.

## Discussion

This study aimed to explore and elucidate the treatment burden experienced by OSA patients who were using CPAP, including but not limited to the burden associated with the device use. Many participants experienced significant workload and burden using CPAP therapy. The most significant aspects of treatment burden (financial burden, problems with equipment, relationship burden and attending healthcare appointments) aligned with reasons for non-adherence to CPAP therapy described in previously published work [13, 14, 23–25]. Most of the interviewees believed that OSA could lead to death through suffocation during sleep, which was one of their strongest motivators for CPAP use. Other reasons for adherence included increased energy levels during the day and fewer interruptions to the partner’s sleep from snoring. However, when the perceived treatment burden exceeded the person’s coping capacity, participants got frustrated, stopped using CPAP therapy regularly and experienced an impaired quality of life.

Acclimatising to the use of CPAP was associated with the greatest treatment burden. Many participants who were unable to get used to the side effects of treatment (e.g. dry mouth), and problems with the CPAP mask (e.g. air leakage and uncomfortable mask fit) reported low motivation in continuing treatment, which resulted in underuse of CPAP therapy. Interestingly, several interviewees initially ceased the use of CPAP but restarted treatment many years later. Their initial decisions to quit CPAP therapy were due to the associated side effects and an uncomfortable mask fit during titration night and first few weeks of treatment. This is similar to the findings of a study conducted in Germany, which found that early experience of treatment-related side effects was the main patient-reported reason for discontinuation of therapy [26]. In the current study, participants who ceased therapy initially remained adherent after restarting therapy because improvements in CPAP design over time resulted in better tolerance of the therapy.

Similar to the results of prior research [13, 27], participants’ relationship quality with bed partners were adversely affected by their OSA. However, for some, relationship quality did not improve after the use of therapy, which could be attributed to reasons such as barriers to intimacy and insecurities regarding their masked appearance, as mentioned by participants in this study. Some interviewees continued sleeping in separate bedrooms with partners despite treatment due to machine-related issues, rendering it uncomfortable for the bed partner. Due to these reasons, some participant’s partners did not support and even discouraged their spouses’ use of CPAP. Contrarily, some participants elicited spousal involvement as a motivator and facilitator for their machine use through providing words of encouragement, reminding the patient to put on the mask and reassuring that they did not look less attractive with the mask. The results of our study suggested that spousal involvement may alleviate or exacerbate the patient-perceived treatment burden depending on the context, aligned with previous research results [13, 28].

This study identified the cost of CPAP as a barrier to use, reflecting the findings of previous studies [5, 12, 29]. Despite receiving government subsidisation for the machine, patients still experienced significant financial burden associated with using CPAP since the subsidy did not cover the cost of the mask and machine replacement parts. Several participants avoided replacing machine parts due to their high cost and continued using worn-out masks and head straps. This increased the chance of air leakage and added to their treatment burden.

The eligibility criteria for government-subsidised CPAP therapy mainly accommodated participants with severe OSA (AHI> 30 events/hour) [30]. Thus, participants in the study who had an AHI between 20 to 30 events/hour were not eligible for a subsidised device even when they experienced daytime sleepiness and significant relationship burden from their OSA. This resulted in a significant financial burden from renting the CPAP machine, and those who were unable to afford continual rental ultimately stopped treatment. Future research could examine the cost-effectiveness of different healthcare funding models for this subgroup of patients.

Routine physician follow-up visits for patients on CPAP therapy were usually performed 6-monthly or once a year in the study hospital outpatient clinic. It appears that study participants waited until their next appointment with a physician to address problems such as CPAP rainout or problems with the headgear. For equipment problems, appointments at a specialist nurse clinic would have been available at short notice in the study hospital, but awareness of this service among the study participants was insufficient, highlighting the need for better communication about available services. Remote monitoring might also help to address some of the struggles that patients experience with CPAP equipment, especially for patients who live far away from the clinic. In line with previous research [13], some participants expressed the need for a CPAP user support hotline. This would enable new CPAP users to obtain immediate advice, rather than waiting for their next face-to-face appointment. New users support programs, which allow new CPAP users to meet experienced CPAP users through peer support groups and group education sessions, have shown an improvement in CPAP adherence compared to usual care [31–33].

Common participant-reported reasons for not exercising included busy schedules and large effort required to exercise, which were similar to results of other studies [34–36]. The low rate of exercise participation in our study could be due to the interviewees’ characteristics. Most participants in this study had co-morbidities such as arthritis and COPD, limiting their ability to perform physical exercises. Surprisingly, in participants who reported significant subjective improvement in chronic fatigue after treatment, exercise participation remained low. This implied that OSA symptoms did not play a major role as a barrier to exercise, reflecting findings from previous literature [37, 38].

This is the first study that evaluated the experience of patients with OSA using a treatment burden framework, i.e. the focus of the study was on all the work that patients must do to care for their health condition(s). Previous studies on treatment burden focused on chronic diseases other than OSA, for example, heart failure and chronic obstructive pulmonary disease [19, 21]. Previous studies that examined the experience of patients with OSA mainly focused on reasons for non-adherence to CPAP therapy without examining other aspects of treatment burden such as prescribed lifestyle changes, attending medical appointments etc. [5, 12].

The study was performed in a public hospital in Sydney, and the results might not be representative of OSA patients treated in a different setting. For example, patients in the private health system might have a different experience than those in the public health system. Doctors might be quicker in responding to patients’ queries, and there might be shorter appointment waiting times, but the financial burden to patients might be increased. The main goal of our study was to explore different areas of treatment burden in patients with OSA, but the emphasis of what constitutes the biggest burden is likely to vary between different settings and health systems. For example, the financial cost associated with CPAP therapy will depend on the insurance system. The results may therefore not be generalisable to other settings. Interviews that were conducted via telephone in our study might have negatively affected rapport and participants’ willingness to discuss private issues [39]. Therefore, it is possible that aspects of relationship burden such as restriction to intimacy were under-reported in phone interviews, and subsequently, the impact of relationship burden might have been underestimated.

## Conclusion

This is the first study exploring all aspects of treatment burden perceived by patients with OSA on CPAP therapy. We found that study participants experienced a significant burden associated with the use of CPAP. Future research could focus on large-scale surveys to quantify the treatment burden elucidated in our study in a representative sample of patients. It is important that physicians explore treatment burden as a possible source of non-adherence, but also enquire about treatment burden in patients who adhere to treatment so that they can provide additional help if necessary.

## Supporting information

S1 Appendix Treatment burden framework for patients with OSA using CPAP. (TIF)

S2 Appendix Participant’s level of treatment burden with different healthcare tasks. (DOCX)

S3 Appendix Participant’s burden level relating to financial and relationship impacts. (DOCX)

S4 Appendix Interview guide developed to explore treatment burden in OSA patients. (DOCX)
