# Supplementary figures and images for: Treatment burden experienced by patients with obstructive sleep apnoea using continuous positive airway pressure therapy
Source: PLoS One. 2021 Jun 7;16(6):e0252915. doi: 10.1371/journal.pone.0252915 (PMC8183990; doi:10.1371/journal.pone.0252915)

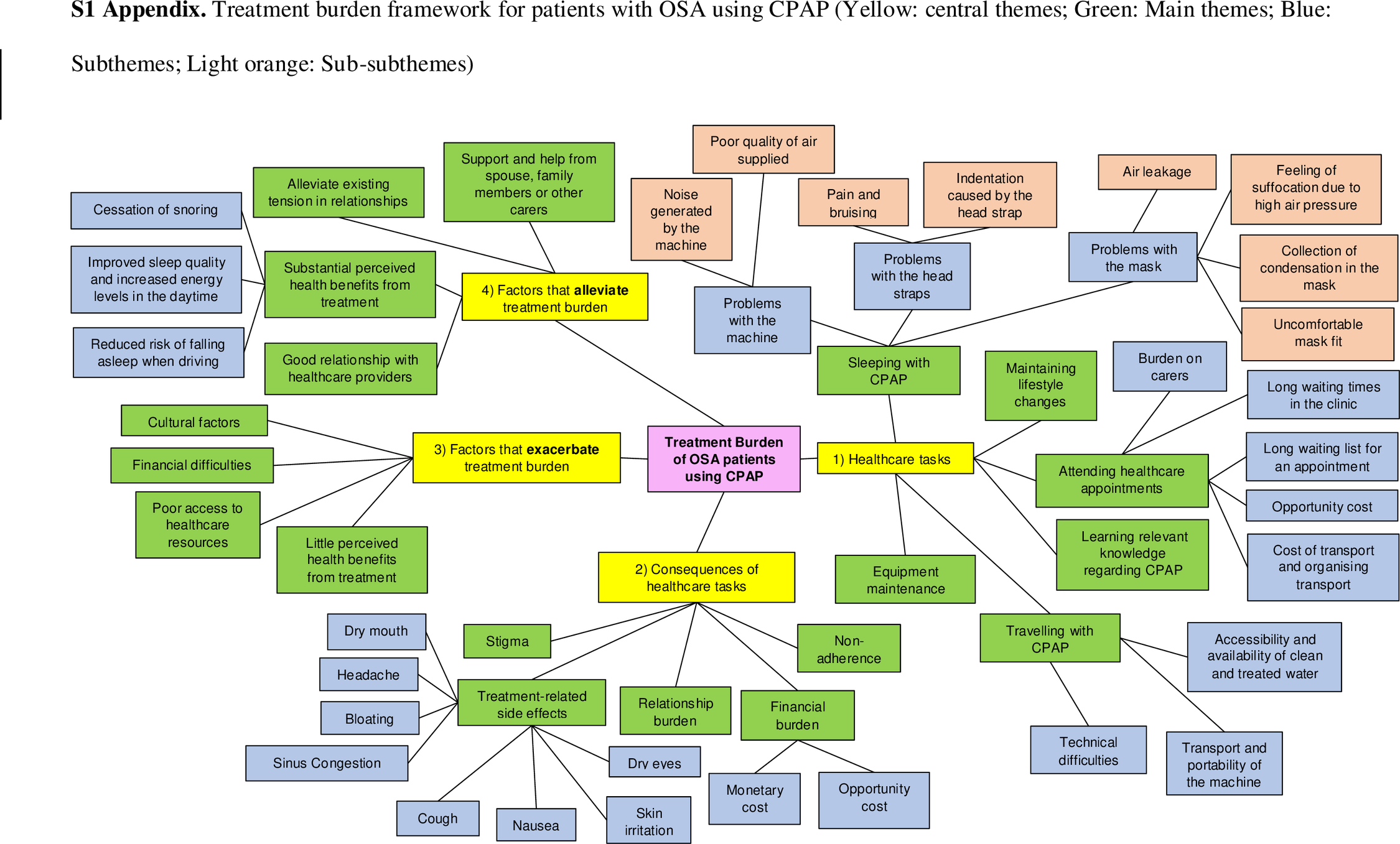

Supplement: S1 Appendix — (TIF) [file pone.0252915.s001.tif]
