# Supplementary material for: Treatment burden experienced by patients with obstructive sleep apnoea using continuous positive airway pressure therapy
Source: PLoS One. 2021 Jun 7;16(6):e0252915. doi: 10.1371/journal.pone.0252915 (PMC8183990; doi:10.1371/journal.pone.0252915)
Supplement: S2 Appendix — (DOCX) [file pone.0252915.s002.docx]

**S2 Appendix.** Participant’s level of treatment burden with different healthcare tasks

|  | | Machine-related tasks | | | | | Machine-unrelated tasks |
| --- | --- | --- | --- | --- | --- | --- | --- |
| Inter-view no. | Patient names*** | Sleeping with CPAP | Equipment maintenance | Attending healthcare appoint-ments | Learning relevant knowledge about CPAP | Travelling with CPAP** | Maintain-ing lifestyle changes |
| 1 | Anna | 1 |  | 3 | 4 | N/A |  |
| 2 | Aaron | 2 |  | 1 | 4 | 1 |  |
| 3 | Belinda | 4 | 4 | 3 | 1 | N/A |  |
| 4 | Charlotte | 4 |  | 1 | 4 |  |  |
| 5 | Chris | 2 |  | 1 | 1 | N/A | 1 |
| 6 | Emily | 4 |  | 4 | 4 | 1 | 4 |
| 7 | Gary | 4 |  | 3 | 1 | N/A |  |
| 8 | Henry | 1 |  | 1 | 3 | 1 | 2 |
| 9 | Heidi | 4 |  | 5 | 5 | N/A | 4 |
| 10 | Isabella | 5 | 4 | 5 |  |  |  |
| 11 | James | 4 | 4 | 3 | 3 | 4 |  |
| 12 | Jessica | 4 | 1 | 3 | 4 | N/A |  |
| 13 | Karen | 5 | 4 | 5 | 3 | 4 | 5 |
| 14 | Luke | 4 | 2 | 3 | 3 | 3 | 3 |
| 15 | Olivia | 4 | 1 | 2 | 4 | N/A |  |
| 16 | Sophie | 5 | 1 | 1 | 4 | N/A | 5 |
| 17 | Stephanie | 2 | 1 | 1 | 1 | N/A |  |
| 18 | Whitney | 2 | 3 | 1 | 1 | 1 |  |
|  | ***Severity grading and its respective colour**** | | | | | | |
|  | Grade 1= |  |  |  |  |  |  |
|  | Grade 2= |  |  |  |  |  |  |
|  | Grade 3= |  |  |  |  |  |  |
|  | Grade 4= |  |  |  |  |  |  |
|  | Grade 5= |  |  |  |  |  |  |

*Boxes are coloured according to the burden severity score (Blanked: topic not discussed; Grade1: light green; Grade 2: dark green; Grade 3: yellow; Grade 4: light red; Grade 5: dark red); **Not every patient had experienced travelling with CPAP; N/A: not applicable; Cells with left diagonal shading meant patient was non-compliant to that healthcare task; ***All participant names are pseudonyms.
