# Supplementary material for: Treatment burden experienced by patients with obstructive sleep apnoea using continuous positive airway pressure therapy
Source: PLoS One. 2021 Jun 7;16(6):e0252915. doi: 10.1371/journal.pone.0252915 (PMC8183990; doi:10.1371/journal.pone.0252915)
Supplement: S3 Appendix — (DOCX) [file pone.0252915.s003.docx]

**S3 Appendix.** Participant’s burden level relating to financial and relationship impacts

| Interview no. | Interviewee’s name* | Financial burden | Relationship burden |
| --- | --- | --- | --- |
| 1 | Anna | 5 | 1 |
| 2 | Aaron | 5 | 1 |
| 3 | Belinda | 3 | 1 |
| 4 | Charlotte | 3 | 1 |
| 5 | Chris | 3 | 4 |
| 6 | Emily | 5 | 1 |
| 7 | Gary | 5 | 1 |
| 8 | Henry | 4 | 3 |
| 9 | Heidi | 5 | 5 |
| 10 | Isabella | 4 | 4 |
| 11 | James | 5 | 4 |
| 12 | Jessica | 3 | 1 |
| 13 | Karen | 5 | 5 |
| 14 | Luke | 3 | 4 |
| 15 | Olivia | 3 | 1 |
| 16 | Sophie | 5 | 1 |
| 17 | Stephanie | 3 | 1 |
| 18 | Whitney | 4 | 1 |
| ***Severity grading and its respective colour***** | | | |
| Grade 1= |  |  |  |
| Grade 2= |  |  |  |
| Grade 3= |  |  |  |
| Grade 4= |  |  |  |
| Grade 5= |  |  |  |

*****All participant names are pseudonyms; **Boxes are coloured according to the burden severity score (Grade1: light green; Grade 2: dark green; Grade 3: yellow; Grade 4: light red; Grade 5: dark red).
