# Supplementary material for: Treatment burden experienced by patients with obstructive sleep apnoea using continuous positive airway pressure therapy
Source: PLoS One. 2021 Jun 7;16(6):e0252915. doi: 10.1371/journal.pone.0252915 (PMC8183990; doi:10.1371/journal.pone.0252915)
Supplement: S4 Appendix — (DOCX) [file pone.0252915.s004.docx]

# **S4 Appendix. Interview Guide developed to explore treatment burden in OSA patients**

| Items | Questions | Probes |
| --- | --- | --- |
| Patient History | - How long have you been diagnosed with Sleep Apnoea? - What symptoms did you experience before you started CPAP? - How was your sleep apnoea diagnosed? - Other than obstructive sleep apnoea, do you have any other health problems? | - Any heart problems, blood pressure, cholesterol problems, or diabetes? |
| Current management of OSA | - What kind of things do you do to manage your apnoea? - How many days a week are you using CPAP? - What do you think is the main issue about the use of the machine? | - Have you been also trying exercising and diet management? How do you find it? - *From a scale of 1-5, with five being the most difficult, how much would you rate the difficulty in making and maintaining these recommended lifestyle changes?* |
| Perceived benefits, disease risks, and information access | - How effective do you think CPAP is? - What is your current understanding of obstructive sleep apnoea? - Where did you get your information about CPAP from? - Have you felt confused about the information given to you? - *From a scale of 1-5, with one being the easiest and five being the most difficult, how much would you rate the difficulty in obtaining clear and up-to-date information about your condition?* | - From word of mouth? Healthcare professionals? Internet? |
| Treatment-related side effects | - Have you experienced any side effects when using CPAP? - Have you experienced any problems with the machine? - (If yes) How have you been overcoming these problems? | - Have you experienced dry eyes, dry mouth, blocked nose, or insomnia when you use your machine? - Problems such as uncomfortable mask fit, and noisy machine? - Problems with the pressure of the machine? |
| Travel-related burden | - Have you travelled abroad with your CPAP? | - Were there any difficulties? How was it managed? |
| Financial burden | - How much does it cost for you to manage your apnoea? This includes gym memberships, diet changes, and the cost of the CPAP machine. - Has managing your OSA affected you financially? - Have you ever thought that CPAP is expensive? - *How reasonably priced do you think CPAP is? Rate it on a scale of 1-5, with one being the most reasonable and five being not reasonable at all.* |  |
| Healthcare access burden | - Have you had any problems with getting an appointment with your CPAP therapist or provider when you need to discuss your concerns or to sort out technical difficulties? - Have you had any difficulties in maintaining doctor appointments? - *From a scale of 1-5, with five being the most burdensome, how burdensome would you rate in maintaining doctor appointments?* | - Have you had any problems with travelling to these healthcare appointments? - Have you had to take time off work to come to these healthcare appointments? |
| Relationship Burden | - Do you have a bed partner? - Has there been any tension in the family, between colleagues and friends caused by your OSA? - Has the use of treatment alleviated these tensions? Or has it worsened these tensions? - Have you ever felt ashamed, embarrassed, or stigmatised when using the machine in front of others? | - What does your partner think about the machine? - Have you made any sleeping arrangements before the use of the machine and after the use of the machine? |
| Factors that exacerbate the perceived treatment burden | - Is there anything else that makes it difficult for you to care for your OSA? |  |
| Factors which alleviates the patient-perceived treatment burden | - Is there anything that makes it easier for you to care for your OSA? - Where do you go for support or help with your machine? - Has your healthcare provider provided enough support for you to use the machine? - How would you describe your relationship with your healthcare provider? - What motivates you to keep using the machine? | - Do you get any support from other people, such as family members and friends? In what ways do they help you? Are they helpful? - If patients mention the fear of stopping breathing as a motivator, then ask: What do you mean by stopping breathing? Do you mean stopping breathing altogether as in death? |
